# Infestation of Transgenic Powdery Mildew-Resistant Wheat by Naturally Occurring Insect Herbivores under Different Environmental Conditions

**DOI:** 10.1371/journal.pone.0022690

**Published:** 2011-07-28

**Authors:** Fernando Álvarez-Alfageme, Simone von Burg, Jörg Romeis

**Affiliations:** 1 Agroscope Reckenholz-Tänikon Research Station ART, Zurich, Switzerland; 2 Institute of Evolutionary Biology and Environmental Studies, University of Zurich, Zurich, Switzerland; Nanjing Agricultural University, China

## Abstract

A concern associated with the growing of genetically modified (GM) crops is that they could adversely affect non-target organisms. We assessed the impact of several transgenic powdery mildew-resistant spring wheat lines on insect herbivores. The GM lines carried either the *Pm3b* gene from hexaploid wheat, which confers race-specific resistance to powdery mildew, or the less specific anti-fungal barley seed chitinase and β-1,3-glucanase. In addition to the non-transformed control lines, several conventional spring wheat varieties and barley and triticale were included for comparison. During two consecutive growing seasons, powdery mildew infection and the abundance of and damage by naturally occurring herbivores were estimated under semi-field conditions in a convertible glasshouse and in the field. Mildew was reduced on the *Pm3b*-transgenic lines but not on the chitinase/glucanase-expressing lines. Abundance of aphids was negatively correlated with powdery mildew in the convertible glasshouse, with Pm3b wheat plants hosting significantly more aphids than their mildew-susceptible controls. In contrast, aphid densities did not differ between GM plants and their non-transformed controls in the field, probably because of low mildew and aphid pressure at this location. Likewise, the GM wheat lines did not affect the abundance of or damage by the herbivores *Oulema melanopus* (L.) and *Chlorops pumilionis* Bjerk. Although a previous study has revealed that some of the GM wheat lines show pleiotropic effects under field conditions, their effect on herbivorous insects appears to be low.

## Introduction

Genetically modified (GM) crops with enhanced resistance to insect pests and/or tolerance to broad-spectrum herbicides were first commercially released in 1996. Since then, the area planted with such GM crops has continuously increased, reaching 148 million hectares in 2010 [1]. Currently, a range of novel GM plants with tolerance to different biotic stresses, with altered nutrition and composition, or with the ability to produce pharmaceuticals are being developed [2]. In addition, a number of crops have been genetically engineered to enhance their resistance to fungal pathogens [3]–[5].

Among the fungal diseases that cause losses in wheat (*Triticum aestivum* L.) worldwide, powdery mildew, *Blumeria graminis* Speer f. sp. *tritici*, is one of the most consistently damaging pathogens [6], [7]. The use of GM wheat plants with enhanced resistance to powdery mildew is being explored as an alternative to the use of chemical fungicides [8]–[11].

One of the concerns associated with the growing of GM crops is that that they could have adverse effects on non-target organisms (i.e., organisms that are not intended to be harmed by the trait under consideration) with potential implications for the sustainable deployment of the crop. Such unintended effects could be caused (i) directly, by the expression products of the inserted genes, (ii) by pleiotrophic effects of the transgene expression, or (iii) by the transgene integration into the plant genome [12], [13].

The aim of our study was to assess the impact of several transgenic disease-resistant wheat lines on the performance of naturally occurring insect herbivores under semi-field and field conditions. We have focused on herbivorous insects as non-target organisms because (i) they are closely associated with the plant and thus are likely to be affected by changes in plant quality, and (ii) because the genetic transformation of the plants should not increase problems with insect pests. We compared the effects of GM and conventional wheat on the abundance of and damage caused by the most common wheat herbivores, namely aphids (Hemiptera: Aphidae), the cereal leaf beetle, *Oulema melanopus* (L.) (Coleoptera: Chrysomelidae), and the chloropid gout fly, *Chlorops pumilionis* Bjerk. (Diptera: Chloropidae).

Two types of GM wheat plants were studied: transgenic lines carrying the *Pm3b* gene from hexaploid wheat, which confers race-specific resistance to powdery mildew [11], and lines that contain the less specific anti-fungal barley seed chitinase and β-1,3-glucanase [10]. While we had no reason to expect a direct effect of the race-specific *Pm3b* transgene product on insects, an adverse effect of chitinase and glucanase could not be excluded because chitin and glucan are main structural components of the exoskeleton of arthropods. In addition to the transgenic lines and their corresponding non-transgenic lines, five conventional wheat varieties as well as barley (*Hordeum vulgare* L.) and triticale (×*Triticosecale* Wittm.) were used for comparison.

The study presented here is part of a multidisciplinary joint project evaluating powdery mildew resistance, field performance, and molecular analysis of transgenic wheat plants and their interactions with the biotic and abiotic environment (http://www.wheat-cluster.ch).

## Materials and Methods

### Plant material

We studied a total of six GM spring wheat lines carrying two different types of resistance genes that confer protection against powdery mildew. Four independent transformation events carrying the transgene *Pm3b* of wheat that provides race-specific resistance to wheat powdery mildew (Pm3b*#1-4*) [14], [15] were used. These lines were generated by biolistic transformation of the spring wheat cultivar Bobwhite SH 98 26, which has no endogenous *Pm3* gene and is, in general, sensitive to powdery mildew [16]. The transgenic Pm3b plants are further described in Brunner et al. [11] and have shown an enhanced resistance against powdery mildew under protected glasshouse conditions [17]. The study also used two transgenic Frisal lines expressing chitinase [Chi (A9)] and chitinase and glucanase [Chi/Glu (A13)] from barley, which should provide a broad active antifungal resistance [18]. However, a previous glasshouse study did not record and enhanced resistance against powdery mildew in these two lines [10]. The null-segregant lines Sb*#1-4* and the Swiss spring variety Frisal were used as the non-transgenic controls of the Pm3b and the Chi (A9) and Chi/Glu (A13) lines, respectively. Additionally, Bobwhite SH 98 26 and the commercial Swiss spring wheat varieties Casana, Fiorina, Rubli, and Toronit, as well as the spring barley variety Estana and the spring triticale variety Trado, all of which are conventional varieties, were used for comparison.

### Convertible glasshouse studies

#### Experimental set-up

Experimental wheat plants were grown during the seasons of 2008 and 2009 in a convertible glasshouse located at the Agroscope Reckenholz-Tänikon Research Station ART in Zurich (Switzerland). The research station is located just outside the city of Zurich in a rural area surrounded by fields, forests, and orchards. The glasshouse provides field-like conditions by exposing the plants to outside environmental temperatures and allowing natural colonization by insects and pathogens. The roof and side walls of the glasshouse automatically opens when weather conditions are good (no rainfall and no strong winds). At night, and in adverse weather conditions, the roof and side walls close automatically [19]. The experiment involved five lines: Pm3b*#1*, Sb*#1*, Chi/Glu (A13), Frisal, and the spring wheat variety Rubli as a conventional control. Experimental wheat plants were grown in 40 plots (80×60×80 cm) arranged in two rows. Each wheat line was replicated eight times, and the replicates were arranged as blocks of five adjacent plots containing the five lines in randomized order. Each plot consisted of a central cylinder (26 cm diameter) for the experimental plants and a surrounding area containing buffer plants (i.e., non-transformed plants of the same variety), simulating a near-field situation. Twenty seeds were planted per central cylinder of each plot and, after seedling emergence, only 10 experimental plants were left to grow. The wheat plants were grown for about 4 months (sowing: April 2 and March 3 in 2008 and 2009, respectively; harvest: July 30 and July 22). Before sowing, basic fertilizer was added to the soil (per plot: 5.8 g P, 7.2 g K, 1.7 g Mg, 8.7 g N in 2008; 10.32 g P, 8 g K in 2009). Additionally, on May 5 and 28 in 2008, and on April 29 in 2009, each plot received 5–7 g of Ammonsalpeter (25% N, 5% Mg, 8.5% S). Plants were watered as needed. Pergamyn-paper bags (Franz Grätzer & Co., Einsiedeln, Switzerland) were placed over individual flowering ears of transgenic and control plants to prevent pollen from escaping the system. In the following, ‘plot’ will be referred to the central cylinder containing the 10 experimental plants.

#### Assessment of powdery mildew infection

Natural powdery mildew infection was scored on each plot for 6 consecutive weeks in 2008 and for 3 consecutive weeks in 2009, starting with the first occurrence of mildew. The Cobb's scale ranging from 0 to 9 (0 = no symptoms, 9 = fully diseased) was used [20]. The area under disease progress curve (AUDPC) was determined [21], [22] with data from 9 and 6 sampling dates in 2008 and 2009, respectively.

#### Monitoring of insect populations and damage

Abundance of aphids and larvae of the cereal leaf beetle *O. melanopus* was estimated by visual counts. Aphids were recorded separately for each species found, i.e., *Metopolophium dirhodum* (Wlk.), *Rhopalosiphum padi* (L.), and *Sitobion avenae* (F.), which are common cereal aphids in Europe [23]. Samplings were conducted weekly between May 8 and July 17 in 2008, and between May 20 and July 9 in 2009. On each sampling date, all the experimental plants from all plots were inspected, and the data were subsequently pooled for each single plot. After *O. melanopus* larvae disappeared in early July, the typical feeding damage was assessed using a modified scheme developed for scoring infection by yellow rust (*Puccinia striiformis* Westend.) [24]. Leaf damage was scored using a scale from 0 to 6 based on the percentage of the leaf surface damaged (0 = no damage, 6 = >75% of the leaf area damaged). For each plant, the flag and the second leaf from two tillers were monitored, and the average damage score was calculated per plot. Plants were also inspected to assess the damage caused by larvae of the chloropid gout fly *C. pumilionis*.

### Field studies

#### Experimental set-up

The field surveys were performed during the 2008 and 2009 growing seasons at Agroscope ART. The field site was located about 300 m from the convertible glasshouse and hence was located in the same rural area. In 2008, the experiment involved 18 different lines: Pm3b*#1–4* and their non-transformed control lines Sb*#1–4*; the transgenic lines Chi (A9) and Chi/Glu (A13) and their control line Frisal; the five conventional spring wheat varieties Bobwhite, Casana, Fiorina, Rubli, and Toronit; the barley variety Estana; and the triticale variety Trado. In 2009, 12 lines were used: Pm3b*#1*–*2* and Sb*#1*–*2*; Chi (A9), Chi/Glu (A13), and Frisal; the conventional varieties Bobwhite, Rubli, and Toronit; barley (Estana); and triticale (Trado).

The plants were tested in a complete randomized block design; in 2008, there were four replications, resulting in a total of 72 plots, and in 2009, there were five replications, resulting in a total of 60 plots. Plots were 3.0×1.3 m in both years. All seeds were treated with the fungicide Jockey (167 g l^−1^ fluquinconazole, 34 g l^−1^ prochloraz; Omya Agro AG, Safenwil, Switzerland) before sowing, and a total of 400 viable seeds m^−2^ were sown in March of each year. In 2009, spreader rows consisting of a 9∶1 mixture of the susceptible varieties FAL94632 and Kanzler were sown between the plots to increase natural powdery mildew infection but no artificial inoculation was made. Fertilizer was administered at a rate of 46 kg P_2_O_5_ ha^−1^ and 60 kg K_2_O ha^−1^ in autumn of 2007 and 2008. Additionally, 30 kg N ha^−1^ was applied in 2008 shortly after sowing and at the phenological stage BBCH 39 (flag leaf stage) [25], [26]. In 2009, the same rate of nitrogen fertilizer was applied the day before sowing and at BBCH 22–29 (two tillers-end of tillering). All plots were sprayed with the herbicide cocktail Concert SX (40% thifensulfurone, 4% metusulfurone-methyl; Stähler Suisse AG, Zofingen, Switzerland) and Starane super (120 g l^−1^ bromoxynil, 120 g l^−1^ Ioxynil, 100 g l^−1^ fluroxypyr-metilheptil-ester; Omya Agro AG, Safenwil, Switzerland) in early May. In both years, experimental plants were harvested in early August.

#### Assessment of powdery mildew infection

Natural powdery mildew infection was determined by estimating the percentage of the leaf surface infected as described above. A total of 20 randomly chosen tillers per plot were inspected every second week, starting in early May. The data for each plot were subsequently pooled. The AUDPC was calculated with data from 5 sampling dates.

#### Monitoring of insect populations and damage

Abundances of aphids and *O. melanopus* larvae were estimated by visual counts. Samplings were conducted every second week between May 6 and July 29 in 2008, and between May 6 and July 14 in 2009. On each sampling date, insect abundance was recorded on the same 20 tillers per plot used for assessing mildew infection, and the data were pooled for each plot. Furthermore, the damage caused by the larvae of *O. melanopus* was determined after larvae had disappeared in early July, using the same scoring scale as in the convertible glasshouse. For each plot, the flag leaf and the second leaf from 20 randomly chosen tillers were monitored, and the average damage score was calculated per plot. Finally, the damage produced by the larvae of the chloropid gout fly *C. pumilionis* was estimated; at the end of the growing season, 30 randomly chosen tillers were checked per plot, and the percentage of damaged plants was determined.

### Data analysis

The data sets from the convertible glasshouse and the field were analysed separately. Natural powdery mildew infection, cumulative numbers of aphids, and cumulative numbers of and damage by *O. melanopus* larvae in the convertible glasshouse were compared using a two-way analysis of variance (ANOVA), with “year” and “wheat line” as fixed experimental factors. For the field data, comparisons between the Pm3b/Sb*#1–2* pairs, and among the transgenic Chi (A9) and Chi/Glu (A13) and their control Frisal plants were conducted using a two-way ANOVA, with “year” and “wheat line” as fixed experimental factors, whereas a one-way ANOVA was used to analyze the Pm3b/Sb*#3–4* pairs, because those lines were only grown in 2008. Field data sets from the different conventional wheat varieties and barley and triticale were also compared using a one-way ANOVA for 2008 and 2009; this was done separately for 2008 and 209 because different wheat varieties were planted in each year. Group mean values were subsequently separated using the Tukey HSD-test. Correlation between aphid abundance and powdery mildew infection and between *O. melanopus* larval abundance and damage was calculated separately in the convertible glasshouse and in the field. Data for aphid abundance and natural powdery mildew infection in the field was square root transformed, and damage by *C. pumilionis* was arcsine transformed to meet the ANOVA assumptions. For all tests, the α-level was set at 5%. Statistical analyses were conducted using the software package Statistica (Version 9.1, StatSoft Inc., Tulsa, OK, USA).

## Results

### Convertible glasshouse studies

#### Powdery mildew infection

**Figure 1:**
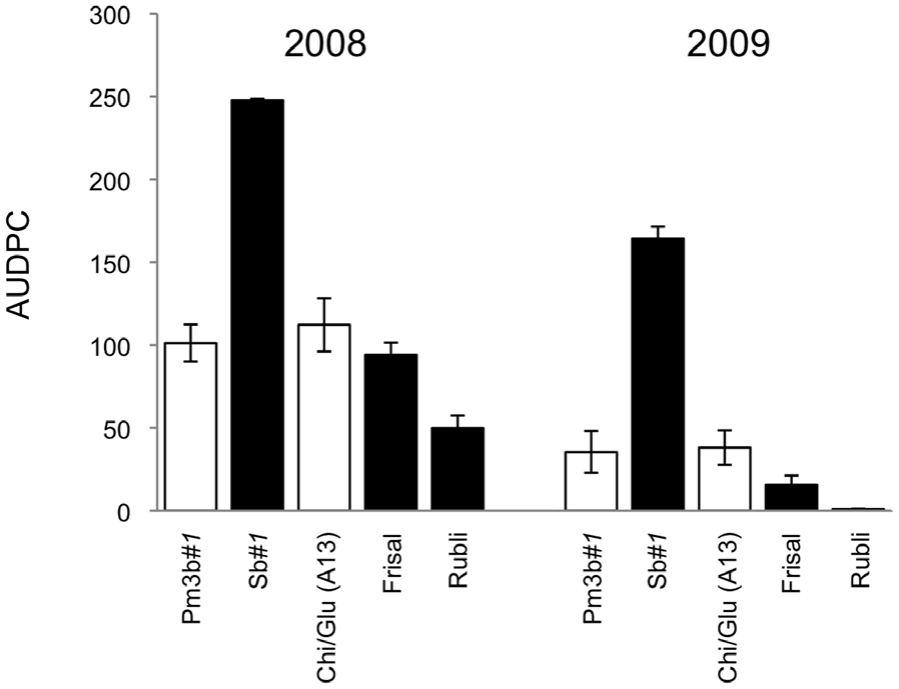
Powdery mildew infection of the wheat plants grown in the convertible glasshouse. Infection was scored using a 0 to 9 scale (0 = no symptoms, 9 = fully diseased). Area under disease progress curve (AUDPC) (±SE) was calculated with data from 9 and 6 sampling dates in 2008 and 2009, respectively. Therefore, AUDPC values are not comparable between years. White bars represent transgenic lines while black bars represent non-transformed lines. (*N* = 8). Significant differences were recorded between the non-transformed line Sb*#1* and the conventional varieties Frisal and Rubli (Tukey HSD test, p<0.001), and between transgenic Pm3b*#1* plants and their control line Sb*#1* over both years of study (Tukey HSD test, p<0.001).

Natural powdery mildew infection in the wheat plants was observed for six consecutive weeks in 2008 and 2009, starting with the first occurrence of mildew. In both study years, significant differences in mildew infection levels were observed among the non-transgenic lines, with Sb*#1* (non-transformed Bobwhite) being the most susceptible by far, followed by Frisal, and Rubli as the least susceptible line (Tukey HSD test, *p*<0.001) (Figure 1). Infection was significantly lower on transgenic Pm3b*#1* plants than on Sb*#1* plants, while infection levels did not differ between Chi/Glu (A13) and its non-transformed Frisal control (Figure 1).

#### Monitoring of insect populations and damage

**Figure 2:**
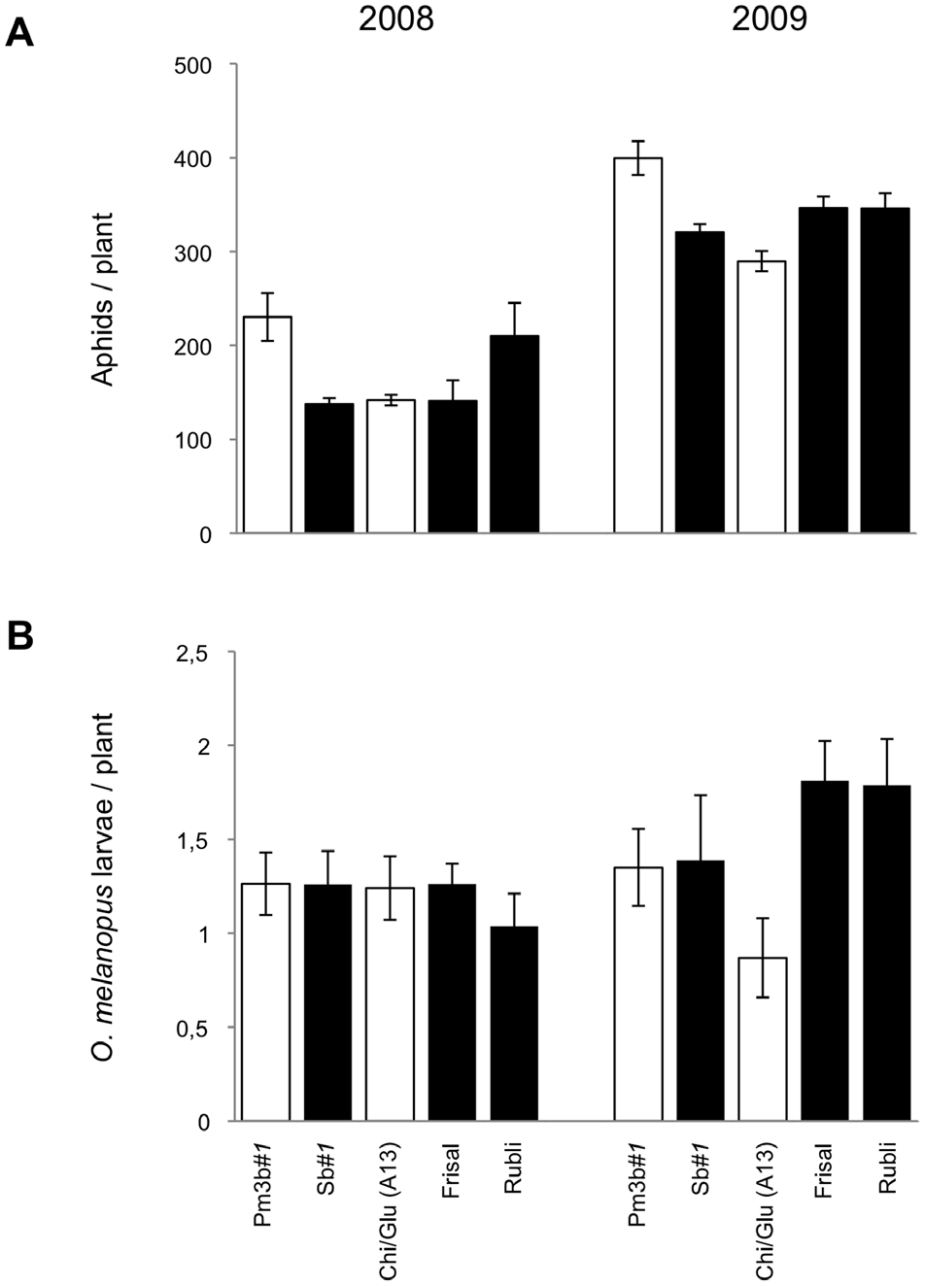
Densities of aphids and *Oulema melanopus* larvae on wheat grown in the convertible glasshouse. Cumulative numbers (± SE) of (A) aphids and (B) *O. melanopus* were calculated with 11 and 9 sampling dates in 2008 and 2009, respectively. White bars represent transgenic lines while black bars represent non-transformed lines. (*N* = 8). For aphid densities, significant differences were recorded between the transgenic Pm3b*#1* and their non-transformed control line Sb*#1* over both years of study (Tukey HSD test, *p* = 0.025).

Abundance of naturally occurring herbivores was estimated by weekly visual counts during two consecutive growing seasons. The cereal aphid species *M. dirhodum*, *R. padi*, and *S. avenae* were found in the convertible glasshouse in both years, although significantly fewer aphids were recorded in 2008 (16.1 aphids/plant) than in 2009 (37.8 aphids/plant). In both years, aphids started to infest the wheat plants by mid-May. Densities then increased to the maximum values of 24.8 aphids/plant in early July 2008 and 151.5 aphids/plant in late June 2009, after which aphid numbers rapidly declined. In both seasons, *M. dirhodum* was the most common species (8.9 and 28.4 aphids/plant in 2008 and 2009, respectively), followed by *R. padi* (6.9 and 7.7 aphids/plant) and *S. avenae* (0.2 and 1.7 aphids/plant). Cumulative numbers (mean ± SE) of aphids/plant recorded on the different wheat lines are shown in Figure 2A. Aphid abundance was similar among the three non-transgenic lines across both years. In contrast, significantly more aphids were recorded on transgenic Pm3b*#1* than on non-transformed Sb*#1* plants (Tukey HSD test, *p* = 0.025). When the three aphid species were analyzed separately, only *M. dirhodum* abundance was significantly different between Pm3b*#1* and Sb*#1* (Tukey HSD test, *p* = 0.034). Across all wheat lines, aphid densities were negatively correlated with powdery mildew infection (*R* = −0.35, *p* = 0.002).

Very low densities of *O. melanopus* larvae were observed in the convertible glasshouse (an average of 0.17 and 0.21 larvae/plant in 2008 and 2009, respectively). In both seasons, cereal leaf beetle larvae were first recorded in mid-May and disappeared by the end of June. Densities reached a maximum of 0.30 and 0.42 larvae/plant in early June 2008 and late May 2009, respectively. Cumulative numbers of *O. melanopus* larvae did not differ significantly among the non-transgenic wheat lines and between transgenic Pm3b*#1* and Chi/Glu (A13) and their respective controls (Figure 2B). Leaf damage caused by *O. melanopus* larvae was significantly higher on Rubli than on Bobwhite and Frisal (Tukey HSD test, p<0.001, and p = 0.039, respectively). Nevertheless, average damage scores translate to leaf surface damage levels <5% in both seasons, indicating that infestation by the beetles was very low.

Damaged produced by larvae of the chloropid gout fly *C. pumilionis* was not observed in the convertible glasshouse in 2008 or 2009.

### Field studies

#### Powdery mildew infection

**Figure 3:**
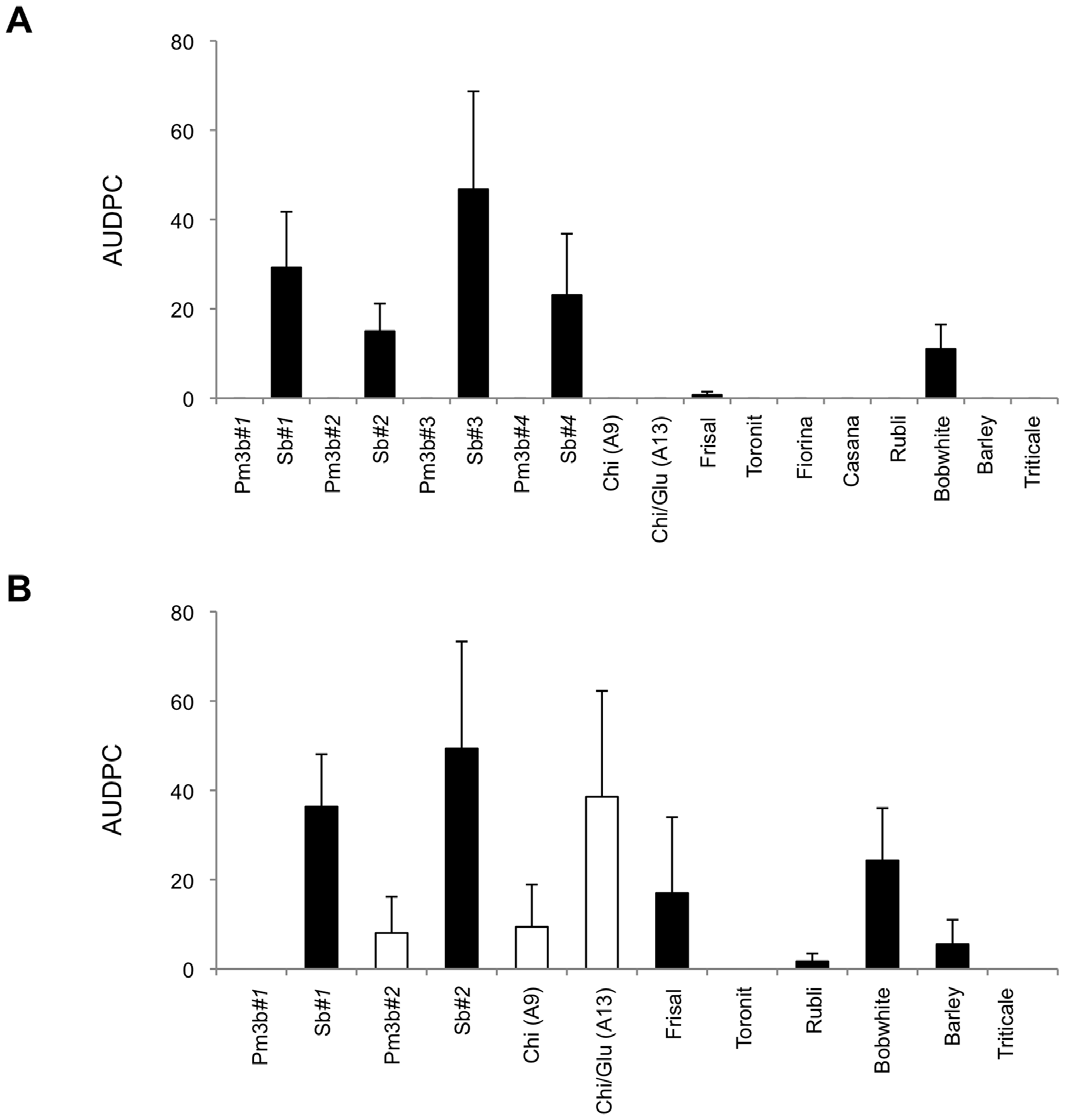
Powdery mildew infection of plants grown in the field. Percentage of the leaf surface infected with powdery mildew was estimated in (A) 2008 and (B) 2009. The area under disease progress curve (AUDPC) (± SE) was calculated with data from 5 sampling dates in both years. White bars represent transgenic lines while black bars represent non-transformed lines. (2008: *N* = 4; 2009: *N* = 5). Significant differences were recorded between Bobwhite plants and the other conventional wheat varieties as well as barley and triticale in 2008 (Tukey HSD test, *p*<0.05). Likewise, significant differences were observed between transgenic Pm3b and their respective non-transformed Sb plants (Pm3b*#1*/Sb*#1*: *F*_1,14_ = 22.7, *p*<0.001; Pm3b*#2*/Sb*#2*: *F*_1,14_ = 6.18, *p* = 0.026; Pm3b*#3*/Sb*#3*: *F*_1,6_ = 11.44, *p* = 0.015, Pm3b*#4*/Sb*#4: F*_1,6_ = 4.53, *p* = 0.077).

Natural powdery mildew infection was determined in wheat, barley, and triticale plants in 2008 and 2009. Low mildew infection levels were observed in the different conventional wheat varieties and in the triticale and barley varieties in both seasons (Figure 3). Infection was higher in Bobwhite plants than in the other conventional wheat varieties or in barley and triticale in 2008 (Tukey HSD test, *p*<0.05), whereas infection levels among conventional plants did not significantly differ in 2009. Powdery mildew infection levels were significantly lower in transgenic Pm3b than in their respective non-transformed Sb plants (Pm3b*#1*/Sb*#1*: *F*_1,14_ = 22.7, *p*<0.001; Pm3b*#2*/Sb*#2*: *F*_1,14_ = 6.18, *p* = 0.026; Pm3b*#3*/Sb*#3*: *F*_1,6_ = 11.44, *p* = 0.015, Pm3b*#4*/Sb*#4: F*_1,6_ = 4.53, *p* = 0.077), whereas infection levels were similar for Chi (A9), Chi/Glu (A13), and non-transformed Frisal plants (Figure 3).

#### Monitoring of insect populations and damage

**Figure 4:**
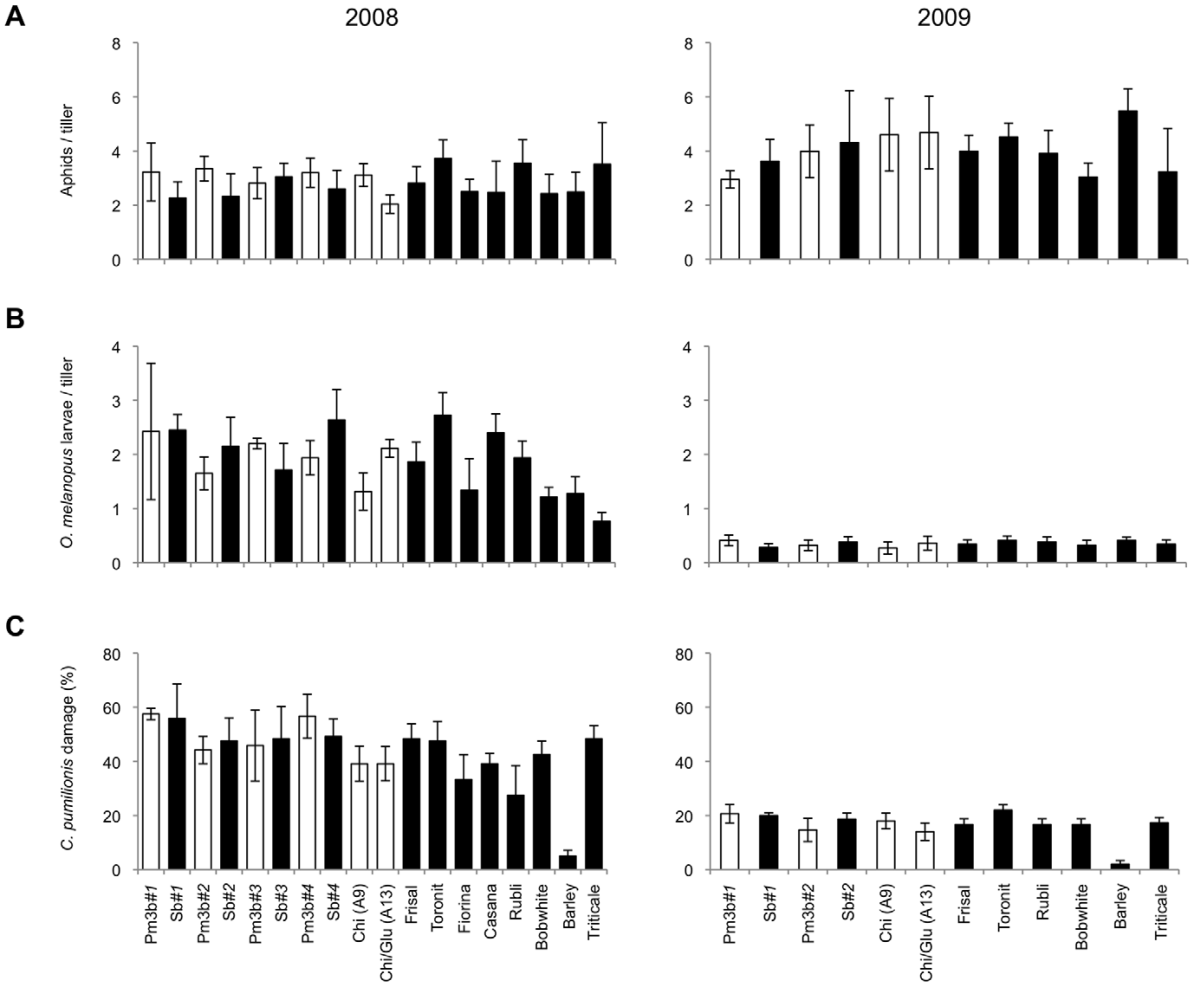
Densities of aphids, *Oulema melanopus* larvae, and damaged by *Chlorops pumilionis* in the field. Cumulative numbers (± SE) of (A) aphids and (B) *O. melanopus* were calculated with data from 7 and 6 sampling dates in 2008 and 2009, respectively, whereas (C) percentage of tillers damaged by *C. pumilionis* was calculated at the end of both growing seasons. White bars represent transgenic lines while black bars represent non-transformed lines. (2008: *N* = 4; 2009: *N* = 5). Significant differences were recorded for *C. pumilionis* larvae between barley plants and the conventional wheat lines and triticale in 2008 and 2009 (Tukey HSD test, *p*<0.05, and p<0.001, respectively).

The abundance of and damage by the most common herbivores were visually estimated in 2008 and 2009. In both growing seasons, the same cereal aphid species were found in the field as in the convertible glasshouse: *M. dirhodum*, *R. padi*, and *S. avenae*. Aphids were first recorded in early May, and their densities subsequently increased to a maximum toward the end of June (1.19 and 2.38 aphids/tiller in 2008 and 2009, respectively). After that, aphid numbers rapidly declined, and by mid-July, very few aphids were observed on the plants. Throughout the season, lower aphid densities were registered in 2008 than in 2009 (0.45 vs. 0.67 aphids/tiller, respectively) (Figure 4A). The most abundant species were *M. dirhodum* (0.27 and 0.30 aphids/tiller in 2008 and 2009) and *S. avenae* (0.15 and 0.37 aphids/tiller), whereas *R. padi* was scarce (0.03 and 0.01 aphids/tiller). Aphid numbers did not significantly differ between the wheat varieties and barley and triticale or between the transgenic plants and their respective controls (Figure 4A). Overall, aphid abundance across all wheat lines was not correlated with powdery mildew infection (*p* = 0.37, *R* = 0.08) in the field.

**Figure 5:**
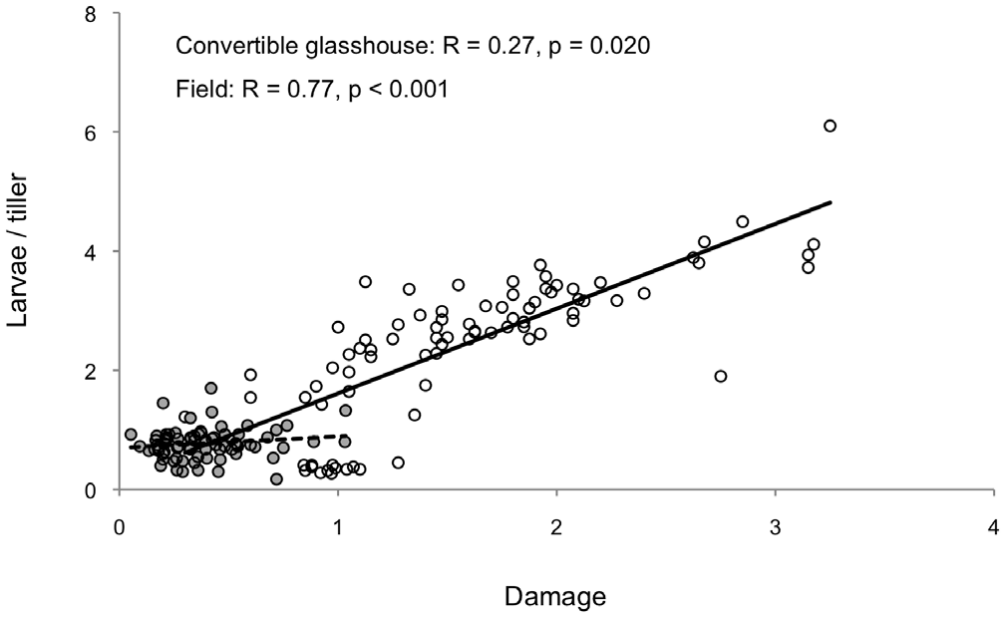
Correlation between abundance per tiller of and damage by *Oulema melanopus* larvae. Data were retrieved from the convertible glasshouse (grey dots) and the field (white dots) in 2008 and 2009.

*Oulema melanopus* larvae were much more abundant in 2008 than in 2009 (0.48 compared to 0.09 larvae/tiller) (Figure 4B). In both growing seasons, densities were highest in early June (0.98 and 0.19 larvae/tiller in 2008 and 2009, respectively), and no larvae were detected by early July. Leaf damage was also higher in 2008 than in 2009, with average scores of 1.7 and 1.0 (scale = 0 to 6), respectively. Leaf surface damage was thus <15% and 5% in the two years. Statistical analysis revealed that neither densities of *O. melanopus* nor the damage caused by the larvae differed significantly among the conventional wheat varieties and barley and triticale plants, or between the transgenic lines and their respective controls (Figure 4B). Damage caused by *O. melanopus* larvae was positively correlated with larval abundance in both the convertible glasshouse and the field (*R* = 0.27, *p*<0.020, and R = 0.77, p<0.001, respectively) (Figure 5).

The damage caused by *C. pumilionis* larvae in the field was much higher in 2008 than in 2009, with 43% and 16% of the tillers being damaged, respectively (Figure 4C). No differences were detected in damage among the different wheat varieties across both seasons, but fewer barley plants than wheat and triticale plants were damaged by *C. pumilionis* in both 2008 and 2009 (Tukey HSD test, *p*<0.05, and p<0.001, respectively). The damage caused by *C. pumilionis* larvae was similar on the transgenic lines and their respective controls (Figure 4C).

## Discussion

Natural powdery mildew infection levels were much higher in the convertible glasshouse than in the field in both years of this study, probably because temperature and humidity were more conducive to mildew development in the glasshouse. Powdery mildew resistance was significantly greater in all Pm3b lines than in their respective non-transformed controls, both in the convertible glasshouse and in the field, which confirmed the findings by Zeller et al. [17] and Brunner et al. [11]. In contrast, the two transgenic lines Chi (A9) and Chi/Glu (A13) did not confer protection against the fungal pathogen when compared to non-transformed Frisal plants, as previously shown by Bieri et al. [10].

In the convertible glasshouse, aphid abundance was negatively correlated with powdery mildew occurrence, and resistant Pm3b*#1* plants harboured larger aphid populations than their susceptible controls. When the three aphid species were analyzed separately, differences were only observed for the most abundant species, *M. dirhodum*, whereas the two other aphid species, *R. padi* and *S. avenae*, remained unaffected. In contrast, aphid densities did not differ between transgenic Chi/Glu (A13) plants and the non-transgenic control variety Frisal, possibly because resistance against powdery mildew was not increased in the transgenic line. The results obtained in our study are consistent with observations in a glasshouse experiment in which the same varieties grown in the convertible glasshouse were artificially inoculated with two different powdery mildew strains [27]. The genetic transformation *per se* does not appear to be responsible for the increased aphid population on the Pm3b*#1* plants. A previous laboratory study demonstrated that different clones of *M. dirhodum* performed similarly on mildew-free Pm3b*#1–4* plants and their Sb*#1–4* controls [28]. The effects on aphids observed in the convertible glasshouse in the present study thus appear to be caused by the lower powdery mildew infection levels on the transgenic Pm3b plants. The mechanisms underlying the negative effects of powdery mildew infection on aphids remain unclear. Reciprocal effects between insects and fungal pathogens sharing the same host have been well documented and can reflect direct interactions between the organisms or indirect interactions that are mediated by the host plant [29]. The effects observed on *M. dirhodum* in our study might be due to the mycelium of the fungal pathogen covering the surface of infected leaves and, therefore, preventing the aphids from penetrating the plant tissue to reach the phloem sap. It is also possible that mildew indirectly affected *M. dirhodum* by inducing physiological changes in the susceptible wheat plants. Fungal infection is associated with a reduced nutrient concentration within host plant tissue [29], and nutrient concentration could also affect phloem composition. In this context, some studies have indicated that the nutritional quality of the phloem sap correlates positively with the performance and behavior of several aphid species [30]–[32]. Hence, future experiments are needed to determine whether phloem sap composition differs between transgenic and control plants in absence and presence of powdery mildew.

In the field, aphid densities and powdery mildew infection were not correlated. Similarly, aphid densities in the field did not differ on the transgenic wheat lines and their respective controls and the conventional wheat varieties. A possible reason for the lack of effects is the fact that both powdery mildew infection and aphid abundance were much lower when compared to the convertible glasshouse.

The abundance of and damage by *O. melanopus* larvae in the convertible glasshouse did not differ on either of the two transgenic lines [Pm3b*#1*, Chi/Glu (A13)] and their respective non-transformed controls. Similarly, *O. melanopus* abundance and damage in the field were similar on the transgenic plants and their controls and among the different conventional wheat varieties and barley and triticale. *O. melanopus* having no preference for particular cereal species including wheat and barley has already been reported by Ventury [33]. It follows that *O. melanopus* also failed to respond to the difference in powdery mildew infection levels between Pm3b*#1* and Sb*#1* plants. The absence of powdery mildew-mediated effects on *O. melanopus* in the convertible glasshouse could be explained by the fact that *O. melanopus* larvae appeared early in the season and completed their life cycle before susceptible plants were highly infected with powdery mildew, or by the fact that larvae preferably feed on younger wheat leaves, including the flag leaf [34], which are much less infected than older leaves. In general, only a few studies have shown that chrysomelid beetles are indirectly affected by fungal pathogens. Performance of larvae and adults of the green dock beetle *Gastrophysa viridula* (De Geer) was reduced when feeding on rust-infected leaves relative to healthy leaves of *Rumex crispus* L. and *Rumex obtusifolius* L. [35]. In that study, the observed effects were related to a lower nutritional quality of the infected leaves. Similarly, developmental time and weight of immature stages of the thistle tortoise beetle *Cassida rubiginosa* Müller were negatively affected when feeding on leaves of *Cirsium arvense* (L.) infected with a necrotrophic fungus [36].

The positive correlation between abundance and damage of *O. melanopus* suggests that the damage caused by *O. melanopus* larvae is a good indicator of their abundance. Because of the positive correlation between abundance and damage of *O. melanopus*, damage assessment could be sufficient to evaluate the effects of transgenic crops on this pest.

In the field experiment, damage caused by the chloropid gout fly, *C. pumilionis*, was similar on transgenic plants and their respective controls, although the damage was lower on barley than on wheat or triticale. A marked preference of the chloropid gout fly for wheat over barley has also been documented by Lilly [37].

The field results obtained here do not reveal any difference in plant–herbivore interactions even though previous studies have revealed that three of the transgenic Pm3b lines (Pm3b*#2*–*4*) showed pleiotropic effects under field conditions, including lower chlorophyll concentration, stomatal conductance, plant height, seed set rates, and spike morphology [11], [17]. These effects were probably due to the fact that the *Pm3b* transgene was overexpressed in the transgenic lines [11]. Interestingly, these effects did not become apparent under controlled glasshouse conditions but only in the field [11], [17]. Biochemical analysis of field collected plant material, however, did not show any difference in the cellulose, hemicelluloses, and lignin content or in the C∶N ratio between transgenic and non-transgenic lines [38]. Similarly, litterbag studies in the field revealed no difference in the decomposition rate of plant material from GM and non-GM wheat lines [39]. Our results are furthermore in agreement with other laboratory, semi-field, and field studies that were performed in this joint project and that detected no impacts of the GM wheat lines on soil microorganisms [40], soil inhabiting annelids [38], or arthropods [28], [41]–[43].

Our study was conducted simultaneously across two growing seasons in a convertible glasshouse and a field located in the same rural area, which allows us to compare both environmental systems. One limitation of the semi-field system (the convertible glasshouse) was that study plants were colonized by only low numbers of certain herbivorous species such as the cereal leaf beetle and the chloropid gout fly. In contrast, wheat plants grown in the convertible glasshouse were more severely infected by powdery mildew and hosted higher aphid densities than plants in the field. For these reasons, effects of powdery mildew infection on aphid densities could only be detected in the semi-field system but not in the field. The convertible glasshouse was also more suitable than the field for analyzing the aphid-parasitoid community structure on the same wheat lines used in our study [42]. This, together with the high financial burden of conducting field experiments with GM plants in Switzerland resulting from government regulatory constraints and public opposition [44], makes the convertible glasshouse a valuable study system for assessing non-target effects of transgenic plants.

Under semi-field conditions, transgenic lines carrying the *Pm3b1* gene that showed an enhanced resistance against powdery mildew where more suitable for aphids when compared to their more pathogen-susceptible control lines. These indirect transformation-related effects, however, were not observed under field conditions. Overall, our study shows that the investigated transgenic wheat lines had a negligible impact on different herbivorous arthropods despite the fact that some of the lines show pleiotrophic effects under field conditions.
